# Characterization of a rare mosaic unbalanced translocation of t(3;12) in a patient with neurodevelopmental disorders

**DOI:** 10.1186/s13039-022-00579-0

**Published:** 2022-03-05

**Authors:** Xiaolin Hu, Elizabeth K. Baker, Jodie Johnson, Stephanie Balow, Loren D. M. Pena, Laura K. Conlin, Qiaoning Guan, Teresa A. Smolarek

**Affiliations:** 1 Division of Human Genetics, Cincinnati Children’s Hospital Medical Center, Cincinnati, OH USA; 2 Department of Pediatrics, University of Cincinnati College of Medicine, 3333 Burnet Ave, Cincinnati, OH USA; 3 Division of Genomic Diagnostics, Children’s Hospital of Philadelphia, Philadelphia, PA USA

**Keywords:** Mitotic rescue, Unbalanced translocation, SNP microarray

## Abstract

**Background:**

Unbalanced translocations may be de novo or inherited from one parent carrying the balanced form and are usually present in all cells. Mosaic unbalanced translocations are extremely rare with a highly variable phenotype depending on the tissue distribution and level of mosaicism. Mosaicism for structural chromosomal abnormalities is clinically challenging for diagnosis and counseling due to the limitation of technical platforms and complex mechanisms, respectively. Here we report a case with a tremendously rare maternally-derived mosaic unbalanced translocation of t(3;12), and we illustrate the unreported complicated mechanism using single nucleotide polymorphism (SNP) array, fluorescence in situ hybridization (FISH), and chromosome analyses.

**Case presentation:**

An 18-year-old female with a history of microcephaly, pervasive developmental disorder, intellectual disability, sensory integration disorder, gastroparesis, and hypotonia presented to our genetics clinic. She had negative karyotype by parental report but no other genetic testing performed previously. SNP microarray analysis revealed a complex genotype including 8.4 Mb terminal mosaic duplication on chromosome 3 (3p26.3->3p26.1) with the distal 5.7 Mb involving two parental haplotypes and the proximal 2.7 Mb involving three parental haplotypes, and a 6.1 Mb terminal mosaic deletion on chromosome 12 (12p13.33->12p13.31) with no evidence for a second haplotype. Adjacent to the mosaic deletion is an interstitial mosaic copy-neutral region of homozygosity (1.9 Mb, 12p13.31). The mother of this individual was confirmed by chromosome analysis and FISH that she carries a balanced translocation, t(3;12)(p26.1;p13.31).

**Conclusion:**

Taken together, the proband, when at the stage of a zygote, likely carried the derivative chromosome 12 from this translocation, and a postzygotic mitotic recombination event occurred between the normal paternal chromosome 12 and maternal derivative chromosome 12 to “correct” the partial 3p trisomy and partial deletion of 12p. To the best of our knowledge, it is the first time to report the mechanism utilizing a combined cytogenetic and cytogenomic approach, and we believe it expands our knowledge of mosaic structural chromosomal disorders and provides new insight into clinical management and genetic counseling.

## Background

Unbalanced translocations resulting in segmental aneuploidy have been reported in approximately 1% of patients with developmental delay and intellectual disability [1, 2]. Unbalanced translocations may be de novo (in about 30% of reported cases) or inherited from one parent carrying the balanced form [3]. Balanced reciprocal translocations may be present in as many as 1 in 500 individuals who are asymptomatic but have an increased risk of infertility, miscarriage, or having children that inherit an unbalanced translocation [4]. Unbalanced translocations are not rare and are usually constitutional and non-mosaic. Cases of mosaic unbalanced translocations are rarely reported, and clinical presentation can be highly variable depending on the chromosomes and genomic material involved, distribution of cells containing the abnormality in the body, and level of the mosaicism. A review of 246 cases with mosaic autosomal structural rearrangements identified 23 cases (11%) with mosaic unbalanced translocations [5]. Most of the non-mosaic cases were de novo, and only a few cases were reported to have the derivative chromosome inherited from a parent carrying the balanced form [6–10]. The molecular mechanisms were not characterized.

We report a case with an extremely rare maternally-derived mosaic unbalanced translocation of t(3;12). We illustrate the complicated mechanism using single nucleotide polymorphism (SNP) array, fluorescence in situ hybridization (FISH), and chromosome analyses.

## Case presentation

An 18-year-old female presented to genetics clinic with a past medical history of microcephaly, short stature, pervasive developmental disorder, moderate intellectual disability, sensory integration disorder, gastroparesis, anxiety, and hypotonia. She was born to a 22-year-old G2P2 female at 40 weeks via vaginal delivery. The pregnancy was uncomplicated until 30 weeks of gestation when an ultrasound revealed intrauterine growth restriction and oligohydramnios. Birth weight was 2097 g (< 2%) and length was 44.5 cm (< 2%). Postnatal karyotype from cord blood revealed 46,XX (parental report). No other genetic testing was performed.

Family history is unremarkable for immediate family members including a healthy 20-year-old biological brother. A maternal aunt had two miscarriages, and a maternal cousin has learning disabilities. Another maternal aunt has two children with craniosynostosis status post-surgery. No other family members have learning or developmental disabilities, short stature, or microcephaly. Parents denied consanguinity.

From a developmental perspective, the proband has global developmental delay. She did not walk until 18 months of age or begin feeding herself until 3.5–4 years old. She had speech delay but now communicates verbally without difficulties. She was followed by developmental pediatrics and received physical therapy, occupational therapy, and speech therapy for global developmental delay from a few months of life until 6 years of age. In addition, she had a neuropsychological evaluation at 11 years old that included the Wechsler Intelligence Scale for Children which revealed a full-scale IQ score < 45. For her anxiety, she has seen a therapist in the past. She is now able to perform most activities of daily living without assistance.

Her physical exam was notable for microcephaly, short stature, and low weight. She was non-dysmorphic. Her neurologic and musculoskeletal exam was significant for an inability to extend her shoulder above 90 degrees; however, she has normal strength and reflexes in her upper and lower extremities. There were no clinical findings that suggested mosaicism, such as differences in skin pigmentation.

Due to her global developmental delay, intellectual disability, and microcephaly, a SNP microarray was ordered to investigate any significant genomic imbalances.

## Methods

**Fig. 1:**
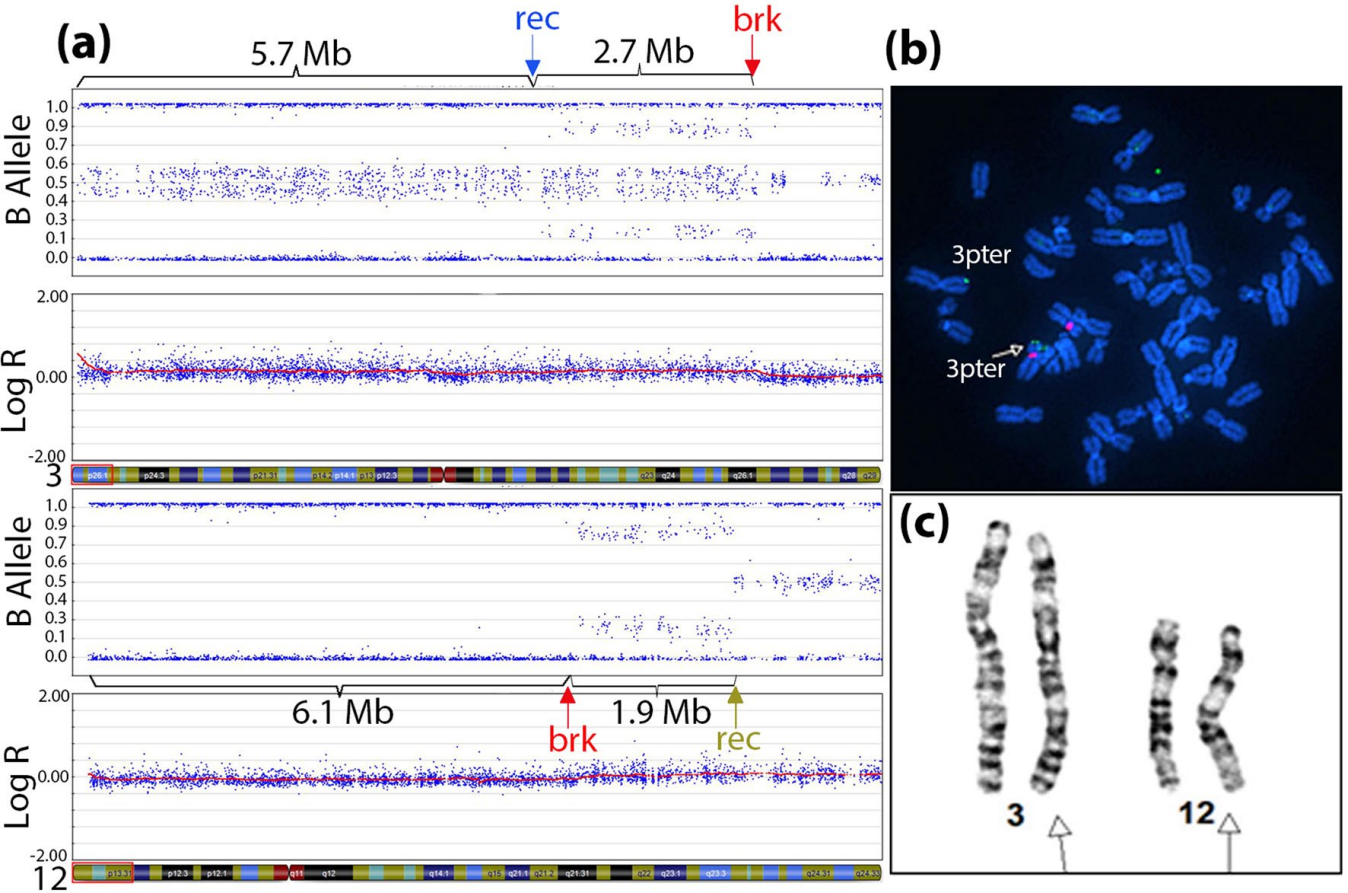
Cytogenetic analysis in the proband and her mother. **a** SNP array analysis in the proband’s peripheral blood showed terminal mosaic duplication of chromosome 3 (3p26.3->3p26.1) on the upper panel and terminal mosaic deletion of chromosome 12 (12p13.33->12p13.31) in the lower panel. brk (in red): breakpoints resulting in derivative 12; rec: meiotic recombination site on chromosome 3 (in blue) and mitotic recombination site on chromosome 12 (in brown). **b** Metaphase FISH analysis in the proband’s peripheral blood detected a subtelomeric signal of 3p (green) translocated to chromosome 12p (visualized by centromere signal shown in red). **c** Chromosome analysis from the proband’s mother’s peripheral blood showed the balanced translocation between chromosome 3 and chromosome 12. Arrows are pointing to the translocated chromosomes

Genomic SNP chromosomal microarray analysis (SNP-CMA) was performed using DNA isolated from uncultured peripheral blood and processed using the Illumina CtyoSNP-850v1.2 BeadChip Platform, which contains approximately 846,500 genome-wide markers. Data was analyzed using Genome Studio v2011.1 (Illumina Inc, San Diego, California). High-resolution chromosome analysis was performed according to standard protocols. Metaphase FISH analysis was used to confirm the results from chromosome analysis. Metaphase cells were hybridized with subtelomeric probes for chromosome 3p (green signal, Abbott Molecular) as well as centromeric probes for chromosome 12 (red signal, Abbott Molecular) (see Fig. 1). FISH images were captured using a Zeiss Axio imager Z2 microscope and analyzed using Applied Imaging software (Cytovision).

## Results

SNP microarray of the proband’s peripheral blood detected an 8.4 Mb terminal mosaic duplication from the short arm of chromosome 3 (3p26.3->3p26.1) (Fig. 1a, upper panel). The duplication contains two parts: the distal part of 5.7 Mb of DNA with two haplotypes, and the proximal part of 2.7 Mb of DNA with three haplotypes. In addition, analysis detected a 6.1 Mb terminal mosaic deletion from chromosome 12p (12p13.33->12p13.31) (Fig. 1a, lower panel). The mosaic deletion also showed a region of homozygosity (ROH), and did not display evidence for a second haplotype in the non-deleted cells. The estimated percentage of the mosaic duplication and deletion are both approximately 25% [11]. Immediately proximal to the mosaic deletion on chromosome 12 (12p13.31) is a 1.9 Mb interstitial mosaic copy neutral ROH. The terminal mosaic duplication on 3p and terminal mosaic deletion on 12p suggest the presence of an unbalanced translocation between chromosome 3p and 12p, der(12)t(3;12)(p26.1;p13.31), in a subset of the patient's cells. Based on the size and genomic content, the mosaic 3p duplication and mosaic 12p deletion were classified as pathogenic. According to ISCN 2020, the microarray karyotype was written as arr[GRCh37] 3p26.3p26.1(61495_5798892)x2-3,3p26.1(5799685_8460083)x2-3,12p13.33p13.31(197841_6250687)x1-2 hmz,12p13.31(6259552_8174984)x2 mos hmz.

To help further delineate the mechanism of the imbalances, high-resolution chromosome analysis was performed. In 35 metaphase cells examined, 25 revealed a normal female karyotype, 46,XX. The remaining 10 showed an abnormal short arm of chromosome 12 consistent with the derivative 12, der(12)t(3;12)(p26.1;p13.31) (data not shown). FISH analysis verified the presence of the abnormal copy of chromosome 12 using probes specific to the subtelomeric region of chromosome 3p and the centromeric region of chromosome 12 on metaphase spreads. Results showed an additional 3p signal on the short arm of a chromosome 12 in three of the 11 cells analyzed (Fig. 1b). These results confirmed the presence of mosaic unbalanced translocation featured as der(12)t(3;12)(p26.1;p13.31). Parental chromosome analysis showed that the father had a normal karyotype, 46,XY, while the mother carried a balanced translocation, t(3;12)(p26.1;p13.31) (Fig. 1c). Incorporating the parental information and FISH results, the proband’s chromosome karyotype was determined to be 46,XX,der(12)t(3;12)(p26.1;p13.31)[10]dmat/46,XX[25].ish der(12)t(3;12)(3PTEL25+ ,CEP 12+)[3].

## Discussion and conclusion

Mosaicism in the presence of an unbalanced translocation is extremely rare. Several possible mechanisms have been proposed including mitotic error, meiotic error followed by postzygotic rescue, and chimerism [8]. Cases that are de novo could be caused by a meiotic or mitotic event between two nonhomologous chromatids followed by the loss of one of the two abnormal cell lines to create mosaicism [12, 13]. Studies have shown that a zygote with an unbalanced rearrangement derived from a balanced parent can lose the abnormal chromosome in a subgroup of cells at an early embryonic stage, whereas the normal homologous chromosome undergoes self-duplication (monosomy rescue) to introduce a normal cell line as well as introducing isodisomy in that chromosome [7, 10]. In other cases, the initial zygote contains three chromosomes due to 3:1 segregation which is followed by an unequal rescue event that generates two cell lines: one with the loss of the abnormal chromosome and the other with the loss of a normal chromosome [6]. Chimerism evolved from two zygotes is another potential mechanism resulting in mosaicism, but this is thought to be extremely rare.

**Fig. 2:**
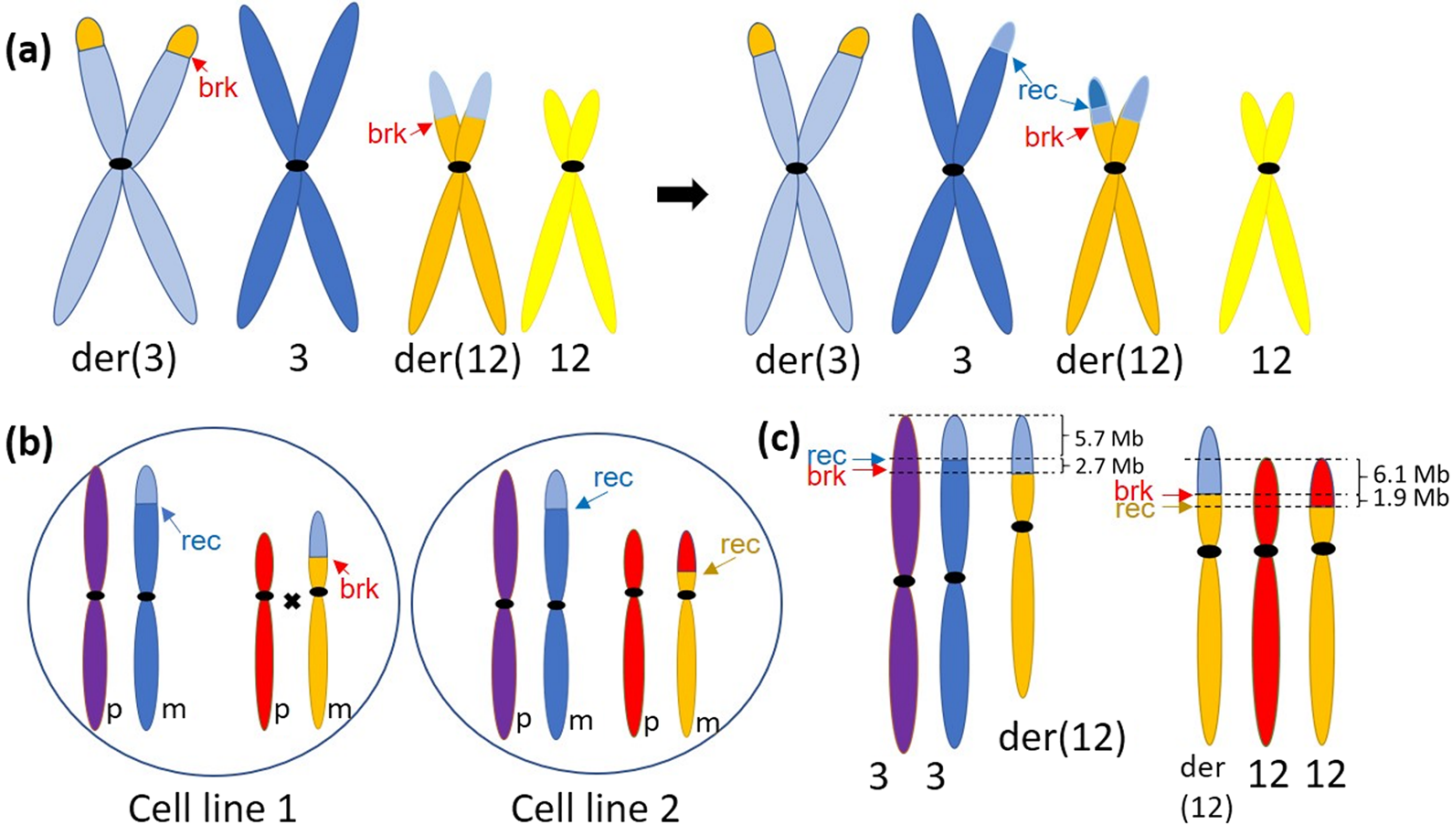
Hypothesized mechanism of the mosaic unbalanced translocation. **a** Left: Balanced translocation between chromosome 3 (light blue) and chromosome 12 (orange) in Mother; Right: Meiotic recombination at chromosome 3p. **b** Cell line 1 contains two normal chromosome 3 (dark blue/light blue from Mom and purple from Dad), the derivative 12 (orange/light blue from Mom) and a normal chromosome 12 (red from Dad). Cell line 2 contains two normal chromosome 3, a paternal normal chromosome 12 (red) and a normal chromosome 12 resulted from mitotic rescue (orange/red). **c** Schematic explanation of the B-allele frequencies in SNP array. Left: Breakpoints are marked to delineate the mosaic duplication on 3p, the 5.7 Mb distal part with two haplotypes (light blue and purple) and the 2.6 Mb proximal part with three haplotypes (light blue, dark blue, and purple); Right: Breakpoints are marked to delineate the 6.1 Mb mosaic deletion on 12p with ROH (red only) and the adjacent 1.9 Mb mosaic copy number neutral ROH region (red and orange)

Previously, we demonstrated the clinical utility of using SNP microarray in detecting rare mosaic chromosomal disorders [11, 14]. Here, we discuss the utility of SNP microarray in determining the complex origin of a mosaic unbalanced translocation. We observed an 8.4 Mb mosaic gain from chromosome 3p and a 6.1 Mb mosaic loss on chromosome 12p. The estimated percentages of both abnormalities were 25% suggesting the coexistence of the two abnormalities in the same cell line. FISH and chromosome analyses confirmed the presence of the der(12)t(3;12)(p26.1;p13.31) in a minor cell line (27.2%-28.6%). Parental chromosome analyses confirmed the translocation was maternally-derived. Furthermore, the complex B-allele frequency patterns of 3p and 12p shown by array analysis suggested additional rearrangements involving the derivative chromosome 12 (Fig. 2) occurred. We hypothesize that during maternal meiosis I, a quadrivalent formed involving the normal chromosome 3, derivative chromosome 3, normal chromosome 12, and derivative chromosome 12 in the primary oocyte. A meiotic recombination event occurred between the normal 3p and the derivative 12 harboring the translocated 3p. The recombination site on der(12) was distal to the breakpoint of the balanced t(3;12) (Fig. 2a). Fertilization of the oocyte with a “normal” recombinant chromosome 3 and der(12) by a sperm containing normal chromosomes 3 and 12 resulted in a zygote with an 8.4 Mb duplication of 3p26.3->3p26.1 (Fig. 2b, cell line 1). The distal 5.7 Mb region of the 3p duplication contains two haplotypes with two copies of the same maternal haplotype and the paternal haplotype, and the proximal 2.7 Mb region of the duplication contains three haplotypes composed of genotypes from the paternal chromosome 3, maternal recombinant 3, and maternal der(12) with 3p translocation. The zygote also contains a 6.1 Mb deletion of 12p (Fig. 2b).

The second cell line with a normal karyotype is thought to arise from a mitotic recombination event that occurred postzygotically between the normal paternal chromosome 12 and the maternal der(12) in an attempt to create two “normal” copies of chromosome 12 (Fig. 2b, cell line 2). The crossover site was 1.9 Mb proximal to the translocation breakpoint on chromosome 12. This mitotic event “corrected” cell line 1 for the partial 3p trisomy and partial deletion of 12p, resulting in mosaic ROH encompassing the entire 6.1 Mb deleted region of 12p in cell line 1 and an adjacent 1.9 Mb mosaic ROH region (Fig. 2b). Figure 2c shows an overall genotype composition of 3p and 12p when considering the two cell lines together explaining the complex B-allele frequency patterns on the SNP array. Compared to previously reported cases, our case proposes a unique mechanism that a mosaic unbalanced translocation originated as an unbalanced rearrangement in a zygote followed by a mitotic recombination event in an attempt to rescue the imbalance. Mitotic recombination has been reported in complex chromosomal rearrangement cases, and it is thought to be driven by low copy repeats and microhomology-mediated break-induced replication [15, 16]. This type of mechanism could cause recurrent complicated rearrangements between homologous chromosomes including the inverted duplication of 8p and triplication of 22q [15, 16]. Whether the mitotic rescue of the der(12) in the current study is mediated by sequencing homology is yet to be determined.

The phenotype of patients with mosaic chromosomal disorders are usually variable. It is difficult to assess what cells contain the imbalances and in what percentages. To our knowledge, no patients with the exact mosaic 3p duplication or mosaic 12p deletion have been reported previously. The DECIPHER disease database lists several reported patients with 3p non-mosaic duplications of similar sizes [17]. Clinical features include hypotonia, skeletal abnormalities, intellectual disability, speech delay, and dysmorphic features (microcephaly, broad forehead, hypertelorism, epicanthus, depressed nasal bridge, and downturned corners of the mouth). Some individuals are reported with smaller duplications including *CHL1, TRNT1, CRBN,* and *CNTN6* from the 3p26.2->3p26.3 region. These patients presented with intellectual disability, developmental delay, epilepsy, autistic features, and behavioral abnormalities [18–20]. For 12p, there are 67 protein-coding genes including 10 genes associated with human disease (*CACNA2D4, CCND2, C12orf4, NDUFA9, KCNA1, KCNA5, WNK1, FGF23, CACNA1C, VWF*). A patient reported with a 6.2 Mb deletion from 12p (12p13.33->12p13.31) presented with intellectual disability, speech delay, anxiety disorder, and psychotic symptoms [21]. A review of 20 cases of patients with deletions of 12p13.33 that included loss of *CACNA1C* identified most individuals with expressive language delay and motor-skill impairment [22]. This phenotype correlates well with our patient who has global developmental delay, moderate intellectual disability, and hypotonia; however, our patient did not have dysmorphic features or psychotic symptoms.

Mosaic chromosomal disorders can have important clinical implications especially in neurodevelopmental disorders. The diagnosis might be underestimated for several reasons. First, the lack of specific clinical phenotype and variable severity pose a diagnostic challenge. Second, somatic mosaicism is easily missed due to the limitation in sampling tissue and detection method [23]. Third, mosaicism may evolve over time with changes in the percentages of normal and abnormal cells [24]. Genome-wide screening methods, such as chromosomal microarray, have been widely used in pediatric genetics and have increased the diagnostic yield for mosaic chromosomal disorders. Here, we provide an example of using combined cytogenetic approaches to resolve an unreported complex etiology of a rare mosaic unbalanced translocation. The presence of the unbalanced translocation in conjunction with the mitotic rescue event resulted in the clinical presentation for this patient. Importantly, we highlight the necessity to perform parental testing on patients with mosaic chromosome disorders, especially in those with unbalanced translocations. Although the mosaicism may be de novo, it could arise from a complex mitotic rescue of the unbalanced rearrangement.

## Data Availability

The authors confirm that the data supporting the findings of this study are available within the article.

## Abbreviations

SNP: Single nucleotide polymorphism
FISH: Fluorescence in situ hybridization
SNP-CMA: Single nucleotide polymorphism chromosomal microarray analysis
ROH: Region of homozygosity
